# First *Candida auris* Outbreak during a COVID-19 Pandemic in a Tertiary-Care Center in Lebanon

**DOI:** 10.3390/pathogens10020157

**Published:** 2021-02-03

**Authors:** Fatima Allaw, Nada Kara Zahreddine, Ahmad Ibrahim, Joseph Tannous, Hussein Taleb, Abdul Rahman Bizri, Ghassan Dbaibo, Souha S. Kanj

**Affiliations:** 1 Division of Infectious Diseases, Internal Medicine Department, American University of Beirut Medical Center, Beirut 1107 2020, Lebanon; fa99@aub.edu.lb (F.A.); ht56@aub.edu.lb (H.T.); ab00@aub.edu.lb (A.R.B.); 2 Infection Control and Prevention Program, American University of Beirut Medical Center, Beirut 1107 2020, Lebanon; nk13@aub.edu.lb (N.K.Z.); ai07@aub.edu.lb (A.I.); jt17@aub.edu.lb (J.T.); 3 Division of Infectious Diseases, Pediatric Department, American University of Beirut Medical Center, Beirut 1107 2020, Lebanon; gdbaibo@aub.edu.lb

**Keywords:** *Candida auris*, outbreak, infection control

## Abstract

*Candida auris* is an emerging fungal pathogen considered as a global health threat. Recently there has been growing concern regarding drug resistance, difficulty in identification, as well as problems with eradication. Although outbreaks have been reported throughout the globe including from several Arab countries, there were no previous reports from Lebanon. We herein report the first cases of *C. auris* infection from the American University of Beirut Medical Center, a tertiary care center in Lebanon describing the clinical features of the affected patients in addition to the infection control investigation and applied interventions to control the outbreak. Fourteen patients with *C. auris* infection/colonization identified using MALDI-TOF and VITEK 2- Compact system were reported over a period of 13 weeks. Patients were admitted to four separate critical care units. All of them came through the emergency room and had comorbid conditions. Half of the patients were infected with COVID-19 prior to isolation of the *C. auris. C. auris* was isolated from blood (two isolates), urine (three isolates), respiratory tract (10 isolates) and skin (one isolate). All the patients had received broad spectrum antibiotics prior to isolation of *C. auris*. Six patients received antifungal treatment, while the remaining eight patients were considered colonized. Environmental cultures were taken from all four units and failed to isolate the organism from any cultured surfaces. A series of interventions were initiated by the Infection Prevention and Control team to contain the outbreak. Rapid detection and reporting of cases are essential to prevent further hospital transmission. A national standardized infection control registry needs to be established to identify widespread colonization.

## 1. Introduction

*Candida auris* (*C. auris*) was first reported as a novel *Candida* species isolated from the ear of a Japanese female patient in 2009 [1]. It is the first fungal pathogen considered as a global health threat that should be reported to local public health authorities [2,3]. *C. auris* exhibits multiple concerning characteristics including multi-drug resistance, difficulty of identification with traditional methods in laboratories and association with health-care outbreaks [4]. It spreads through person-to-person transmission in hospital settings, thus differs from other *Candida* species that usually arise from the patient’s own microbiome and are part of the gastrointestinal flora [5]. It shares the same virulence factors with other *Candida* species, but it also can evade innate immunity and form biofilms that are resistant to all antifungal agents [6,7,8]. Its occurrence can range from colonization to invasive disease and candidemia with a high mortality rate reaching 68% [9]. Over the past 10 years it has been reported worldwide in more than 35 countries, including nine from the Middle East [2]. According to the current theory, four different clades have emerged spontaneously in different areas around the world; these are the East Asian, the South Asian, the African and the South American. A possible fifth clade was later identified in Iran [10]. In the Middle East, the first case was reported in Kuwait in 2014 [11,12,13] with subsequent reports from Israel [14], Oman [15,16], Saudi Arabia [17,18], United Arab Emirates [19], Iran [10,20], Sudan [21] and Qatar [22].

In this series, we report the first *Candida auris* outbreak in Lebanon occurring at the American University of Beirut Medical Center (AUBMC), a tertiary care hospital acting as a referral center for patients from across Lebanon as well as neighboring countries. We describe the clinical features of the 14 affected patients in four critical care units, in addition to the infection control interventions that were initiated to contain the outbreak.

## 2. Cases Description

### 2.1. Laboratory Methods

It is crucial to identify *C. auris* for adequate treatment. In fact, it can be misidentified as *C. haemulonii* if regular commercial laboratory tools were used [23]. In 2016, MS-VITEK matrix-assisted laser desorption ionization time of flight mass spectrometry (MS-VITEK MALDI) was efficiently used to identify *C. auris* [24].

Cultures were requested by physicians to direct antifungal treatment of symptomatic patients. The requested clinical samples were blood, Deep Tracheal Aspirates (DTA) and urine.

Surveillance samples were requested by the infection controlpreventionist to detect skin colonization in 26 asymptomatic patients who might have been potentially exposed. Samples were taken from nares, axilla and groin as well as rectal swabs. 

All clinical and surveillance isolates were cultured on Sabouraud Dextrose Agar and incubated at 35–37 °C between 48 h and up to 2 weeks. Identification of the fungal strains was conducted by MALDI-TOF and then validated by VITEK 2-Compact system. Genome sequencing and molecular analysis are not available at our center. Samples were referred for future analysis. 

### 2.2. Patients’ Demographics and Clinical Stay 

We reported 14 patients with *C. auris* infection and colonization in our hospital from October 2020 until end of December 2020. The index case started in our Neurology Intensive Care Unit (Neuro-ICU) and consequently, the 13 remaining cases were reported afterwards within a period of 3 months. 

The first patient was a 75-year-old man with advanced cutaneous T cell lymphoma who was initially receiving treatment as an outpatient at our center. His disease was non-responsive to multiple lines of chemotherapy regimens. He presented to the Emergency Department (ED) at the beginning of October with pneumonia and respiratory failure and then was admitted to the Neuro-ICU. Fever work-up showed *C. auris* in urine after one week of hospital stay. No antifungal treatment was initiated because there were no signs or symptoms of urinary tract infection. He died 10 days later because of respiratory failure.

Subsequently, 13 patients with *C. auris* were identified. Seven of which had an underlying malignancy and 7 had prior COVID-19 pneumonia. Thirteen out of the 14 patients were intubated and mechanically ventilated. The organism was cultured from different sites: blood (2 isolates), urine (3 isolates), respiratory tract (10 isolates) and skin (1 isolate). Six patients received antifungal treatment while the remaining 8 cases were considered to be colonized. Five patients passed away. Table 1 shows the demographics of the patients identified with *C. auris.*

Susceptibility tests were requested against amphotericin B, caspofungin, fluconazole, and micafungin using the Etest antimicrobial susceptibility test (bioMérieux SA, Marcy-l’-Etoile, France). We used the Centers for Disease Control and Prevention (CDC) tentative cut-off values to interpret strains’ susceptibilities based on expert opinion [2]. Table 2 shows the available susceptibility profiles of the *C. auris* isolates. 

Resistance to antifungal drugs was defined as such: fluconazole ≥32 µg/mL, amphotericin B ≥2 µg/mL, caspofungin ≥2 µg/mL, and ≥2 µg/mL for micafungin were used [2]. Our isolates showed susceptibility to caspofungin and micafungin, and resistance to fluconazole and amphotericin B.

Three patients received directed therapy with caspofungin for candidemia, while four others received empiric treatment with an echinocandin (anidulafungin or caspofungin) as they were considered at high risk for developing invasive fungal infection. The remaining patients did not receive any antifungal treatment as the *C. auris* was considered as a colonization without signs of active infection, or the patients were managed as palliative care, or were discharged home prior to the identification of *C. auris*. 

### 2.3. Outbreak Timeline

**Figure 1:**
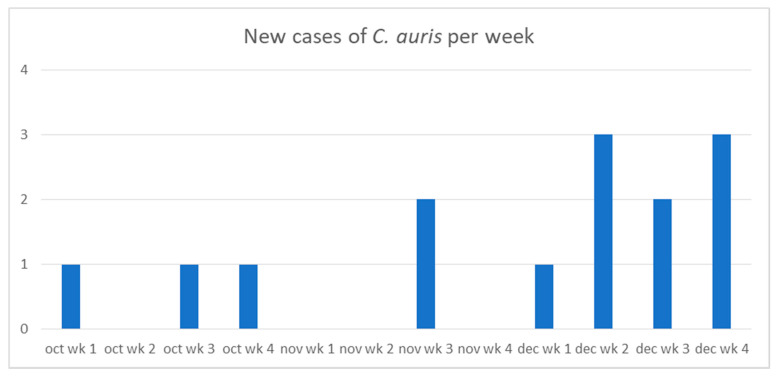
Incidence of *C. auris* per week.

**Figure 2:**
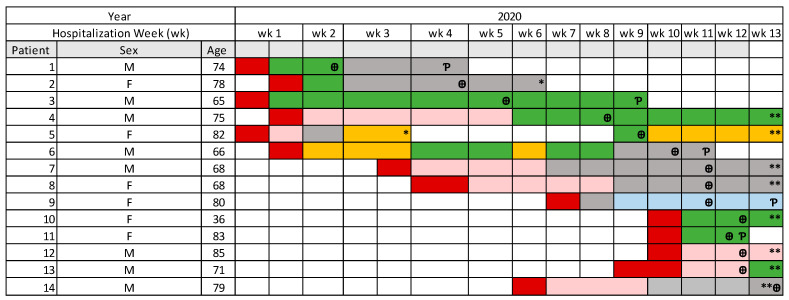
Patients’ locations and transfers within the hospital in relation to time. Red, ED; Green, Neuro-ICU; Grey, ICU; Yellow, RCU; Pink, COVID ICU; Blue, medical/surgical ward; *, Discharged; **, Still admitted; **Ƥ**, passed away; **Ꚛ**, Week of the diagnosis; M, Male; F, Female.

The first case of *C. auris* was identified on 7 October 2020 and cases continued to emerge over a period of 13 weeks. The graph in Figure 1 shows the timeline of new cases over the 13 weeks. These patients were admitted from the emergency department (ED) to different critical care units: Intensive Care Unit (ICU), Neuro-ICU, Respiratory Care Unit (RCU) and COVID-Intensive Care Unit (COVID-ICU) before acquiring *C. auris*. Some of the patients moved from one ICU to another before diagnosis. Neuro-ICU was a common location for 9 out of the 14 patients. Seven out of the 14 patients were admitted to COVID-ICU located in a separate building than the other 3 ICUs, and acquired *C. auris* while in COVID-ICU (2 patients) or following transfer to other critical care units for continuity of care (5 patients). Figure 2 shows patient’s location and transfer within the hospital in relation to the date of *C. auris* diagnosis at the time of the outbreak. All 14 patients underwent Computed Tomography (CT) scans in addition to daily chest X-Rays using the portable machines. Bedside dialysis was done to 5 out of the 14 patients. Other invasive or interventional procedures were done to some of these patients such as bronchoscopy, gastroscopy, and CT guided interventions. All these procedures were done while the cleaning and disinfection of the machines were considered suboptimal because of delay in identifying *C. auris* in patients undergoing procedures. 

### 2.4. Infection Control Interventions

Standard precautions measures are routinely applied to all admitted patients in our medical center. During the COVID-19 pandemic, all healthcare were required to wear facemasks as soon as entering the medical facility. In addition, the patients were asked to wear a facemask when a healthcare worker enters the room. COVID-19 patients are placed under contact and droplet isolation when admitted to the regular COVID-19 unit and under airborne and contact isolation when admitted to COVID-19 ICU. When ICU patients are boarding in the emergency department for lack of bed availability, a High-Efficiency Particulate Air (HEPA) filter is used when negative pressure rooms are not available.

Following the identification of the first case of *C. auris*, the Infection Prevention and Control (IPC) team initiated an investigation and implemented a series of interventions that included isolation precautions, multidisciplinary meetings, education on IPC measures as well as environmental measures to contain the spread of *C. auris*.

Detailed directives were communicated to the medical, nursing and auxiliary teams assigned to the care of patients with *C. auris* to prevent the transmission of *C. auris* to other patients, staff and the hospital environment, as shown in Table 3.

Additional IPC interventions were applied following the reporting of subsequent *C. auris* cases by the microbiology laboratory. These included environmental cultures taken from the direct patient environment and equipment. Skin screening from all patients admitted to critical care units during the same period were also obtained. Those samples were collected from high touch areas and shared items and machines. Sterile swabs were moistened with sterile saline using isotonic solution (0.9%) [2].

All environmental cultures were reported as negative. One patient grew *C. auris* from skin screening. All patients were bathed with 4% Chlorohexidine to promote skin decolonization, as shown effective by the CDC [2].

The initial delay in reporting *C. auris* by the microbiology laboratory might have played a role in the spread of the pathogen to other patients before implementing rigid infection control measures. When the first *C. auris* was isolated, the result was suspected, and it was sent to a reference laboratory abroad for confirmation. This contributed to a delay in the initial reporting of *C. auris*. In addition, some clinical samples yielded polymicrobial growth, consequently, subculturing was required to isolate and identify pure colonies, which required additional time.

## 3. Discussion

In a relatively short period of time, *C. auris* has become one of the most feared pathogens in healthcare settings. Despite increased awareness, numerous outbreaks have been recently reported from many countries including Arab countries in the Middle East. The first regional case was reported in Kuwait in 2014 [11], followed by several other reports from the region [12,13,14,15,16,17,18,19,20,21,22]. To our knowledge, this is the first *C. auris* report from Lebanon. To date, we have identified 14 cases in our critical care units within a period of 13 weeks. The index case was the first patient admitted to Neuro-ICU through ED. This patient had no previous hospitalization and no recent travel history, suggesting a community source, although community spread is rare. So far it is unclear whether *C. auris* was a long-term colonizer or recently introduced from the environment [25]. From the subsequent 13 cases identified, seven had been admitted to the same unit during the course of their treatment. This unusual transmission between patients in Neuro-ICU was likely caused by the delay to report *C. auris*, lack of proper hand washing, inadequate cleaning and disinfection of the patient equipment such as ventilators and direct environment. The current COVID-19 epidemic in the country entailed a tremendous pressure at our center suffering currently from full capacity in critical care units and in ED. Such pressure resulted in suboptimal application of infection control measures in ED. Additionally, all ventilators remain continuously occupied making it impossible to culture for *C. auris,* contrary to a study done in Oman where swabs collected from a ventilator in two different beds in the ICU were positive for *C. auris* [16] A case-series from UK showed that the transmission of *C. auris* was associated with reusable axillary temperature probes [26], whereas the latter did not grow any candida species at our center. In an intensive care unit in India, transmission through hands of health care workers was shown to be responsible for the rapid spread of *C. auris* [27]. Intense training was scheduled for all nursing and inhalation staff at our center. 

Moreover, the outbreak in our ICU might have been caused by two different scenarios. The transfer of one patient from Neuro-ICU to ICU might have caused the emergence of the ICU cases. The other scenario might be related to the transfer of a patient from COVID-ICU to ICU. In fact, two patients were identified with *C. auris* during their stay in COVID-ICU after being admitted from the ED. The environmental contamination of the ED remains a possibility suggesting either cross-contamination of equipment such as CT, X-ray machines or ventilators.

*C. auris* was isolated from 13 patients after a prolonged hospital stay. All patients received broad spectrum antibiotics including piperacillin-tazobactam, carbapenems and ceftolozane-tazobactam. Interestingly, seven out of the 14 patients (50%) had severe COVID-19 illness, a finding that might be related to the rise of fungal infections during the COVID-19 pandemic [28]. Twelve out of 14 patients received steroids, all of them had central venous catheters and Foley catheters, and 13 out of the 14 were mechanically ventilated. All these factors were previously established as risk factors in several case-series [29,30]. Moreover, two patients had cutaneous T-cell lymphoma with multiple skin lesions which might have contributed to a loss in skin integrity and *C. auris* invasion. 

There are no official guidelines for the management of *C. auris* infection. Usually, echinocandins are the first line therapy [31]. Our available results showed low MIC to echinocandins and high MIC to fluconazole, which is similar to the results from Middle Eastern countries except for Iran where it might have been attributed to a potential fifth clade. Moreover, MIC to Amphotericin B varied among the cases, a pattern that was observed in six counties of the Middle East [32]. 

Resistance has been reported against echinocandins. One of our patients in whom we lacked susceptibility results died after three days of candidemia, despite having used anidulafungin. In fact, combination therapy with voriconazole and micafungin has been suggested for in vitro synergistic activity [33]. Liposomal Amphotericin B has also been used in case of unresponsiveness to echinocandins [11]. In addition, repeat cultures and susceptibility tests are recommended during treatment in order to identify possible emergence of resistance [34].

Antifungal susceptibilities vary according to different geographic locations and therefore it is recommended to identify the antifungal susceptibilities of all isolates in order to guide our therapy. Our analysis lacked susceptibility tests on several of the patients because of financial issues in view of the current economic crisis in Lebanon. However all isolates are saved for future microbiology tests and sequencing to determine the *C. auris* clade. 

In the Middle East, *C. auris* has been reported in six countries so far. Infection with *C. auris* is likely underreported due to the lack of adequate laboratory tools, especially in low-income countries. Our analysis lacked genomic sequencing due to the economic and health difficulties Lebanon is going through. This made it impossible to identify the clade of our outbreak and relate our strains to other outbreaks. This work will be done in the future to determine the clade of our outbreak and relate our strains to other regional outbreaks. Genomic sequencing shows that different clades exhibit different virulence genes [35], which might affect phenotypic characteristics of *C. auris* and its ability to aggregate in the environment and form biofilms [36]. Whether these differences are related to the ability to cause nosocomial outbreaks is yet to be determined. 

According to the European Center for Disease Control (ECDC), institutions should investigate the possibility of an outbreak even when a single case has been identified [37]. Thus, we started a thorough investigation to determine the mechanism and the sequence of transmission in our institution. 

Rigorous IPC measures were implemented in the critical care units to prevent further spread. The newly-applied IPC measures are ongoing even though *C. auris* was not recovered from all the reusable items of medical equipment that were cultured. 

It is vital to use the appropriate laboratory methods to promptly identify *C. auris* in hospital settings. This will guide appropriate antifungal treatment for patients as well as implement infection control interventions such as strict isolation, active surveillance of potentially exposed contacts and rigorous cleaning and disinfection of the patient’s equipment and the environment using chlorine-based solutions. Prompt implementation of evidence-based infection control interventions to limit the spread of *C. auris* plays a major role in limiting further transmissions. All isolates have been saved for future studies. In addition, comparing the clades to the *C. auris* isolated from Lebanon to isolates from other countries in the region will shed light on the potential origin of the outbreak.

## 4. Conclusions 

Our report highlights the emerging threat of *C. auris* resulting in rapid transmission in healthcare settings. The outbreak occurred in the midst of the COVID-19 pandemic. The spread is alarming despite rigorous infection control interventions. Rapid detection and reporting of cases are essential. Many microbiological laboratories in Lebanon and the region do not routinely speciate non-*albicans Candida* spp. There is a need for a national and a regional surveillance program to better understand the scope of *C. auris* infection, assess the clade relatedness and guide the implementation of rigorous ICP strategies to curtail the spread of this pathogen in the region.

## Figures and Tables

**Table 1:** Clinical characteristics of the patients with positive cultures for *C. auris.*

Cases	1	2	3	4	5 *	6	7	8 *	9	10 *	11	12	13	14
**Sex/Age**	M/74	F/78	M/65	M/75	F/82	M/66	M/68	F/68	F/80	M/71	F/83	F/36	M/85	M/79
**Site of isolation**	Urine	DTA	DTA	DTA + Urine+	DTA	Blood	DTA	DTA	Urine	DTA	Skin	DTA	DTA	DTA
				Blood										
**ICU stay before isolation in days**	6	18	31	40	26	62	50	40	28	15	12	10	10	48
**Date of isolation**	7/10	22/10	29/10	19/11	22/11	5/12	8/12	8/12	12/12	17/12	17/12	18/12	18/12	30/12
				27/11										
				23/12										
**Medical condition**	Cutaneous T cell lymphoma	Small bowel obstruction	Brain abscess	COVID-19 ARDS/metastatic prostate cancer	COPD/Respiratory failure/	Metastatic esophageal cancer	COVID-19 ARDS	COVID-19 ARDS	Achalasia/malignant ascites	Cutaneous T cell lymphoma in remission/COVID-19 ARDS	COPD	Diffuse large B cell lymphoma in relapse	COVID-19 ARDS	COVID-19 ARDS CLL
			(fusobacterium)		COVID-19									
			pituitary macroadenoma											
**Intubated/ventilated**	Yes	Yes	Yes	Yes	Yes	Yes	Yes	Yes	No	Yes	Yes	Yes	Yes	Yes
**Indwelling urinary catheter**	Yes	Yes	Yes	Yes	Yes	Yes	Yes	Yes	No	Yes	Yes	Yes	Yes	Yes
**Presence of CVC**	Yes	Yes	Yes	Yes	No	Yes	Yes	Yes	Yes	Yes	Yes	Yes	Yes	Yes
**Broad spectrum antibiotics**	Yes	Yes	Yes	Yes	Yes	Yes	Yes	Yes	Yes	Yes	Yes	Yes	Yes	Yes
**Previous anti-fungal**	Yes	No	Yes	Yes	Yes	Yes	Yes	Yes	Yes	Yes	Yes	Yes	Yes	Yes
**Steroid Intake**	Yes	No	Yes	Yes	Yes	Yes	Yes	Yes	Yes	Yes	Yes	No	Yes	Yes
**Treatment provided**	No	No	No	Yes	No	Yes	No	Yes	Yes	Yes	No	Yes	Yes	No
**Outcome/disposition**	Died of hypoxic respiratory failure	Discharged home	Died of septic shock	Still in Neuro-	Still in RCU	Died of septic shock	Still in ICU	Still in ICU	Died of septic shock	Still in Neuro-ICU	Died of septic shock	Still in ICU	Still in COVID-ICU	Still in ICU
				ICU										

M, male; F, female; DTA, deep tracheal aspirate; COPD, chronic obstructive pulmonary disease; CLL, chronic lymphocytic leukemia; CVC, central venous catheter; ARDS, acute respiratory distress syndrome, ICU, intensive care unit; RCU, respiratory care unit; Neuro-ICU, neurology intensive care unit; *, Cases tested for *C. auris* susceptibilities.

**Table 2:** The minimal inhibitory concentration (MIC) of *C. auris* isolates against five antifungal agents from three of the patients.

Anti-fungal agents MIC	Case 5 *	Case 8 *	Case 10 *
Amphotericin B	2	4	8
Caspofungin	0.25	0.25	0.25
Fluconazole	32	32	32
Micafungin	≤0.06	0.12	≤0.06
Voriconazole	0.25	≤0.12	0.25

* Cases from Table 1 tested for *C. auris* susceptibilities.

**Table 3:** Infection control measures implemented in the units having patients with *C. auris*.

Infection Control Measures	
Antisepsis/Cleaning and disinfection	Hand Hygiene protocol was changed: Staff were asked to wash their hands with antiseptic soap and water followed by alcohol-based hand rub when exiting rooms of patients with *C. auris*
	Disinfectants wipes or solutions containing quaternary ammonium compounds were withdrawn from the units since they have poor activity against *C. auris*: A sporicidal solution was introduced to clean/disinfect all patient related medical equipment Sodium hypochlorite diluted at 10% was used for cleaning and disinfection of floors and surfaces of patients’ rooms (during patient stay and upon discharge) Frequency of cleaning/disinfecting high touched surfaces was increased to every 2 h Terminal air decontamination using Hydrogen peroxide was used upon discharge of patients
Visitors’ restrictions	Visitors were restricted from entering the patient’s rooms: Education and training sessions were given to the necessary companions Video calls replaced the actual visits during the patients’ stay
Patients	Isolation Precautions: Contact precautions were initiated upon reporting of *C. auris* by the laboratory Impermeable gowns and gloves became mandatory before entry to the patient’s room Antiseptic soap was provided inside the patients’ rooms (4% chlorhexidine) Universal face masks were already in use due the COVID-19 pandemic. Similarly, eye protection/face shield when performing aerosol generating procedures were already implemented All patients admitted to the critical care units were bathed using 4% chlorhexidine solutions Sharing of equipment between patients identified with *C. auris* and other patients was forbidden
Screening/Environmental cultures	Thirty-four environmental cultures were taken from the following sites: Direct patient environment, nursing stations, portable X-Ray machine, 4 dialysis machines, bilevel positive airway pressure machine, ventilators, temperature probes, computers, dispensers for personal protective equipment, medication trolleys.
	Skin screening was taken from 26 patients who were admitted during same period of stay of the 13 *C. auris* patients Samples were taken from nares, axilla and groin as well as rectal swabs
Results	*C. auris* was not recovered from any of the 34 environmental cultures Only one patient out of the 26 screened patients grew *C. auris* from skin screening

## Data Availability

The data presented in this study are available on request from the corresponding author. The data are not publicly available due to privacy.
